# **pared**: model selection using multi-objective optimization

**DOI:** 10.1093/bioadv/vbag211

**Published:** 2026-08-19

**Authors:** Priyam Das, Sarah Robinson, Christine B Peterson

**Affiliations:** Department of Biostatistics, Virginia Commonwealth University, Richmond, VA 23219, United States; Department of Statistics, Rice University, Houston, TX 77005, United States; Department of Statistics, Rice University, Houston, TX 77005, United States

## Abstract

**Motivation:**

Model selection is a ubiquitous challenge in statistics. For penalized models, model selection typically entails tuning hyperparameters to maximize a measure of fit or minimize out-of-sample prediction error. However, these criteria fail to reflect other desirable characteristics, such as model sparsity, interpretability, or smoothness.

**Results:**

We present the R package pared for Pareto-based model selection using multi-objective optimization. The package provides a generic interface, pared_optimize(), that allows users to define model-specific tuning parameters, objective functions, and objective directions. Built-in wrappers are also provided for the elastic net, fused lasso, fused graphical lasso, and group graphical lasso. Gaussian process-based multi-objective optimization is used to approximate the Pareto front, and interactive graphics allow users to inspect trade-offs among fit, sparsity, smoothness, structural similarity, and other user-defined criteria.

**Availability and implementation:**

The pared R package and vignettes are available at https://github.com/priyamdas2/pared. The archived release corresponding to this manuscript is available on Zenodo with DOI: 10.5281/zenodo.20533535.

## 1 Introduction

Model selection is a classic and well-studied statistical problem. It is a non-trivial challenge to select a model that balances the trade-off of model parsimony, fit, and generalizability. To paint a picture of this challenge in the high-dimensional setting, we begin by considering the lasso (Tibshirani 1996), as a classic penalized regression approach with a single penalty parameter. The choice of this penalty parameter, typically denoted λ, is critical, as the value of λ determines both the estimated coefficient values as well as the model sparsity. Typically, λ is chosen using cross-validation, which utilizes data-splitting to estimate prediction error on a held-out test set (Wu and Wang 2020). Given a grid of λ options, the optimal value is selected as the one that minimizes the out-of-sample prediction error.

Although simple, parameter selection via cross-validation has several key drawbacks. Firstly, it can lead to instability, as different random splits of the data will lead to different selected values of the parameters (Roberts and Nowak 2014). Secondly, in practice, it may result in models that include more features than desirable, as the optimization is focused around prediction accuracy rather than sparsity. Finally, it may be time-consuming to re-fit the model for all the values on the grid. This may be particularly inefficient if the grid contains many values that are far away from the optimum. As an additional challenge, prediction error on a test set is not a sensible target for unsupervised learning problems such as clustering or graphical model inference (Li *et al*. 2013b).

As an alternative, fit criteria may be applied for model selection. Classical measures of fit include the information criteria, most notably AIC (Akaike 1974) and BIC (Schwarz 1978). These may be applied to penalized regression models, but lack theoretical support for the p>n setting and tend to select overly dense models (Zou *et al*. 2007). Another vein of research relies on stability under resampling as a criteria for model selection (Liu *et al*. 2010, Meinshausen and Bühlmann 2010). However, this approach inherits limitations of the base learner: e.g. the lasso tends to select only one from among a set of correlated features. This may result in low stability for correlated predictors with lasso as the base model.

The problem becomes even more challenging in models that require the choice of multiple hyperparameters. In particular, regression models that seek to address limitations of the lasso may incorporate more complex penalty structures with multiple tuning parameters. For example, the elastic net (Zou *et al*. 2007) and fused lasso (Tibshirani *et al*. 2005), which target smoothness or grouping of the coefficients in addition to sparsity, require the choice of two tuning parameters. In the graphical modeling framework, the graphical lasso relies on a single $\ell_{1}$ penalty to achieve sparsity in the precision matrix (Friedman *et al*. 2008). Methods designed for the inference of multiple networks, including the fused and group graphical lasso (Danaher *et al*. 2014), incorporate an additional penalty term to encourage sharing of common edges or similar edge values across multiple networks.

Critically, these methods seek to achieve multiple desired objectives, including sparsity and some version of smoothness or similarity. Relying on a single measure of model fit for model selection neglects these desiderata. Pareto optimization, also known as multi-objective optimization, seeks to identify a set of solutions that lie along the Pareto front. These points represent optimal trade-offs in the sense that no criterion can be improved without sacrificing at least one other criterion. This perspective offers a useful conceptual shift for statistical model selection: rather than forcing model choice through a single scalar criterion, it treats fit, sparsity, smoothness, and structural similarity as competing scientific objectives whose trade-offs should be made explicit.

Building on this idea, we introduce the pared R package for **Pare**to-optimal **d**ecision making in model selection. The contribution of pared is two-fold: conceptually, it promotes Pareto-front exploration as a practical framework for statistical model selection; computationally, it provides a modular R software implementation that connects existing Gaussian process-based multi-objective optimization tools with user-defined model-selection workflows. The package also includes convenience wrappers for common penalized regression and graphical-model settings. The name pared reflects the goal of helping users pare or trim model complexity to identify solutions that represent reasonable trade-offs among fit, sparsity, smoothness, and other model-specific criteria.

## 2 Methods

The pared package supports Pareto-based model selection when model choice depends on multiple, potentially competing criteria. We first describe the software architecture, then summarize the Pareto optimization workflow, and finally describe the built-in wrappers and their default objectives.

### 2.1 Software architecture

The pared package is organized around a generic multi-objective optimization engine, pared_optimize(), together with model-specific convenience wrappers and visualization tools. The central idea is to separate the statistical model from the Pareto optimization routine: users provide a function that maps tuning parameters to objective values, along with parameter bounds, objective names, and objective directions (e.g. to minimize or maximize). These objectives may represent model-specific criteria such as prediction error, deviance, information criteria, model size, coefficient shrinkage, graph sparsity, smoothness, cross-group similarity, or computational cost. The generic interface also records whether each objective is to be minimized or maximized and passes user-specified solver options through a control argument. The optimization details are described in the next subsection.

For user-defined problems, the core interface can be called directly. In the example below, objective_fun evaluates the model-selection criteria, lower and upper define the tuning-parameter bounds, objective_directions specifies which objectives are minimized or maximized, and control passes solver options. Complete runnable examples, including user-defined objectives and objective-space constraints, are provided in the associated GitHub vignette.

res <- pared_optimize(

 objective_fun = my_objective,

 lower = c(-2, -4),

 upper = c(3, 1),

 objective_directions = c(“min”, “min”, “max”),

 control = list set (...)

)

### 2.2 Pareto optimization workflow

We next describe how the generic call above is translated into a Pareto-based model-selection problem.

**Pareto-based model selection.** Let $x\in X\subset R^{d}$ denote a vector of tuning parameters, and let


$$g(x)=\{g_{1}(x),\ldots ,g_{q}(x)\}$$

denote model-selection objectives. A configuration $x_{a}$ dominates $x_{b}$ if it is at least as good in all objectives and strictly better in at least one objective, after placing all objectives on a common minimization scale. The non-dominated configurations define the estimated Pareto set, and their objective values define the estimated Pareto front.

Here, x may contain penalty parameters, while g(x) may contain measures of fit, sparsity, smoothness, structural similarity, or other model-specific criteria. In most applications, no single tuning configuration optimizes all objectives simultaneously. The purpose of pared is therefore to approximate the Pareto set, allowing users to inspect the resulting trade-offs among competing model-selection goals (Collette and Siarry 2003).

Multi-objective hyperparameter optimization has been studied extensively in the machine-learning and optimization literature, including Bayesian optimization (Snoek *et al*. 2012), evolutionary algorithms (Karl *et al*. 2023), and particle-swarm methods (Xue *et al*. 2013). In pared, we use the R package GPareto to approximate Pareto fronts when objective evaluations are computationally expensive. This allows pared to leverage existing Gaussian process-based multi-objective optimization while focusing on the statistical model-selection interface.

Operationally, pared evaluates the user-supplied objective function at an initial set of tuning configurations, fits Gaussian process surrogate models to the objectives through GPareto, and sequentially proposes additional configurations to improve the estimated Pareto front. Objectives declared as maximization targets are internally sign-reversed for optimization and then returned on their original scale for reporting and visualization. After the optimization budget is exhausted, pared returns the estimated non-dominated configurations, their objective values, and interactive 2D or 3D projections of the Pareto front. Objective-space preferences can also be incorporated by modifying the user-defined objective function, e.g. by penalizing candidate models that exceed a prespecified support size, runtime, or complexity threshold; a constrained-objective example is provided in the associated GitHub vignette.

### 2.3 Built-in wrappers and default objectives

In addition to the generic pared_optimize() interface, pared includes convenience functions for several commonly used penalized regression and graphical-model procedures: the elastic net, fused lasso, fused graphical lasso, and group graphical lasso. These functions make standard workflows easy to use while retaining the more general user-defined interface described above. We briefly summarize the supported models below; additional notation and implementation details are provided in Section 1, available as supplementary data at *Bioinformatics Advances* online.

For regression problems, let $X\in R^{n\times p}$ denote the design matrix, $y\in R^{n}$ the response vector, and $\beta \in R^{p}$ the regression coefficient vector. The elastic net (Zou and Hastie 2005) combines $\ell_{1}$ and $\ell_{2}$ penalties and is estimated by solving


(1) $$\hat{\beta }=\underset{\beta }{\text{argmin}}(\frac{1}{2n}||y-X\beta |{|}^{2}+\lambda [\alpha ||\beta |{|}_{1}+(1-\alpha )||\beta |{|}^{2}]).$$

where λ controls overall regularization and α controls the balance between lasso and ridge penalties. The fused lasso (Tibshirani *et al*. 2005) is estimated by solving a penalized least-squares problem that adds a penalty on successive coefficient differences,


(2) $$\hat{\beta }=\underset{\beta }{\text{argmin}}(\frac{1}{2n}||y-X\beta |{|}^{2}+\lambda_{1}||\beta |{|}_{1}+\lambda_{2}\sum_{j=2}^{p}|\beta_{j}-\beta_{j-1}|).$$

where $\lambda_{1}$ controls sparsity and $\lambda_{2}$ controls smoothness across ordered coefficients. For these regression wrappers, the fitted model is obtained for each candidate tuning-parameter value, and pared then evaluates multiple model-selection objectives for the resulting coefficient estimate. As summarized in Table 1, pared_ENet() uses deviance to measure fit, the number of nonzero coefficients to measure sparsity, and the $\ell_{2}$ norm of β to measure coefficient shrinkage. Similarly, pared_FLasso() uses residual sum of squares (RSS), the number of nonzero coefficients, and coefficient roughness (i.e. mean absolute difference of consecutive betas), defined through successive differences in β. These objectives reflect common trade-offs in penalized regression: improved fit may require additional variables, while stronger shrinkage or smoother coefficient profiles may improve interpretability and reduce overfitting. They are default choices rather than restrictions; alternative regression objectives can be supplied through the generic pared_optimize() interface.

**Table 1:** Built-in pared wrappers and default objectives.^a^

Wrapper	Tuning parameters	Fit	Parsimony	Structure/regularity
pared_ENet()	α,λ	Deviance (Min)	Nonzero coefficients (Min)	$\ell_{2}$ norm (Min)
pared_FLasso()	$\lambda_{1},\lambda_{2}$	RSS (Min)	Nonzero coefficients (Min)	Roughness (Min)
pared_JGL(fused)	$\lambda_{1},\lambda_{2}$	AIC (Min)	Total edges (Min)	Precision MAD (Min)
pared_JGL(group)	$\lambda_{1},\lambda_{2}$	AIC (Min)	Total edges (Min)	Shared edges (Max)

a “Min” and “Max” indicate whether the objective is minimized or maximized.

For graphical-model problems, pared supports the joint graphical lasso (Danaher *et al*. 2014). Let *K* denote the number of groups, $n_{k}$ the sample size in group *k*, $S^{\left(k\right)}$ the empirical covariance matrix, and $\Theta^{\left(k\right)}$ the group-specific precision matrix. The joint graphical lasso estimates ${\left\{\Theta^{\left(k\right)}\right\}}_{k=1}^{K}$ by maximizing


(3) $$\{\hat{\Theta }\}=\underset{\left\{\Theta \right\}}{\text{argmax}}(\sum_{k=1}^{K}n_{k}[\text{log}\;\text{det}(\Theta^{\left(k\right)})-\text{tr}(S^{\left(k\right)}\Theta^{\left(k\right)})]-P(\{\Theta^{\left(k\right)}\})),$$

over positive-definite precision matrices. There are two variants, defined by the choice of penalty function *P*. The fused graphical lasso encourages similar edge values across groups:


$$P(\{\Theta \})=\lambda_{1}\sum_{k=1}^{K}\sum_{i\neq j}|\theta_{ij}^{\left(k\right)}|+\lambda_{2}\sum_{k<k^{\prime }}\sum_{i,j}|\theta_{ij}^{\left(k\right)}-\theta_{ij}^{\left(k^{\prime }\right)}|.$$

The group graphical lasso encourages overlapping edge selection across groups:


$$P(\{\Theta \})=\lambda_{1}\sum_{k=1}^{K}\sum_{i\neq j}|\theta_{ij}^{\left(k\right)}|+\lambda_{2}\sum_{i\neq j}{\left(\sum_{k=1}^{K}{\left(\theta_{ij}^{\left(k\right)}\right)}^{2}\right)}^{1/2}.$$

In both variants, $\lambda_{1}$ controls within-group sparsity, while $\lambda_{2}$ controls cross-group similarity. For these graphical-model wrappers, pared evaluates model-selection objectives after fitting the joint graphical lasso at each candidate pair $\left(\lambda_{1},\lambda_{2}\right)$. As summarized in Table 1, pared_JGL() with method = ‘fused’ uses AIC to measure fit, the total number of selected edges across groups to measure sparsity, and the mean absolute difference of the estimated precision matrices to measure cross-group similarity. For pared_JGL() with method = ‘group’, the first two objectives remain AIC and total number of selected edges, while the third objective is the number of shared edges across groups, which is maximized to favor common network structure. These objectives reflect the intended trade-off in joint network estimation: a model with lower AIC may be denser, whereas a sparser or more strongly shared network may be easier to interpret and compare across groups. As with the regression wrappers, these are default choices; alternative graphical-model objectives can be supplied through pared_optimize().

Taken together, the built-in wrappers provide reproducible default templates for common penalized regression and graphical-model workflows, while the generic pared_optimize() interface allows these objectives to be modified or replaced in user-defined applications. For all wrappers, pared returns the estimated Pareto-optimal tuning configurations and interactive visualizations that allow users to inspect trade-offs among the selected criteria. Complete runnable examples are provided in the GitHub vignettes, and *in silico* evaluations with known ground truth are provided in Sections 2 and 3, available as supplementary data at *Bioinformatics Advances* online.

## 3 Illustrative analyses

In this section, we illustrate pared through both controlled *in silico* studies with known ground truth and a real proteomic network analysis.

### 3.1 *In silico* validation

We first evaluated pared using two *in silico* studies, with full details provided in Section 2, available as supplementary data at *Bioinformatics Advances* online. In an elastic-net variable-selection simulation, pared identified Pareto-front models that retained competitive prediction performance while exposing sparser alternatives to conventional cross-validation and EBIC-based tuning; the full results are reported in Table 1, available as supplementary data at *Bioinformatics Advances* online. In a group graphical lasso simulation with known precision-matrix structure, the sparse pared representative reduced false-positive edges relative to an AIC-selected GGL baseline while improving edge-recovery F1; the full results are reported in Table 2, available as supplementary data at *Bioinformatics Advances* online. These simulations are motivated by the fact that pared returns a Pareto set rather than a single automatically selected model. The goal is therefore to assess whether the returned front contains practically useful solutions under known truth. The results show that the Pareto set includes sparse representatives with competitive fit and improved selection accuracy, supporting its use for identifying scientifically reasonable trade-offs. Representative computation times for vignette-scale examples are also reported in Table 3, available as supplementary data at *Bioinformatics Advances* online. These include pared_ENet, pared_FLasso, and pared_JGL under both fused and group penalties, and provide practical guidance on runtime as a function of model class, sample size, dimension, and the user-specified Pareto_budget.

### 3.2 TCPA proteomic network analysis

**Figure 1:**
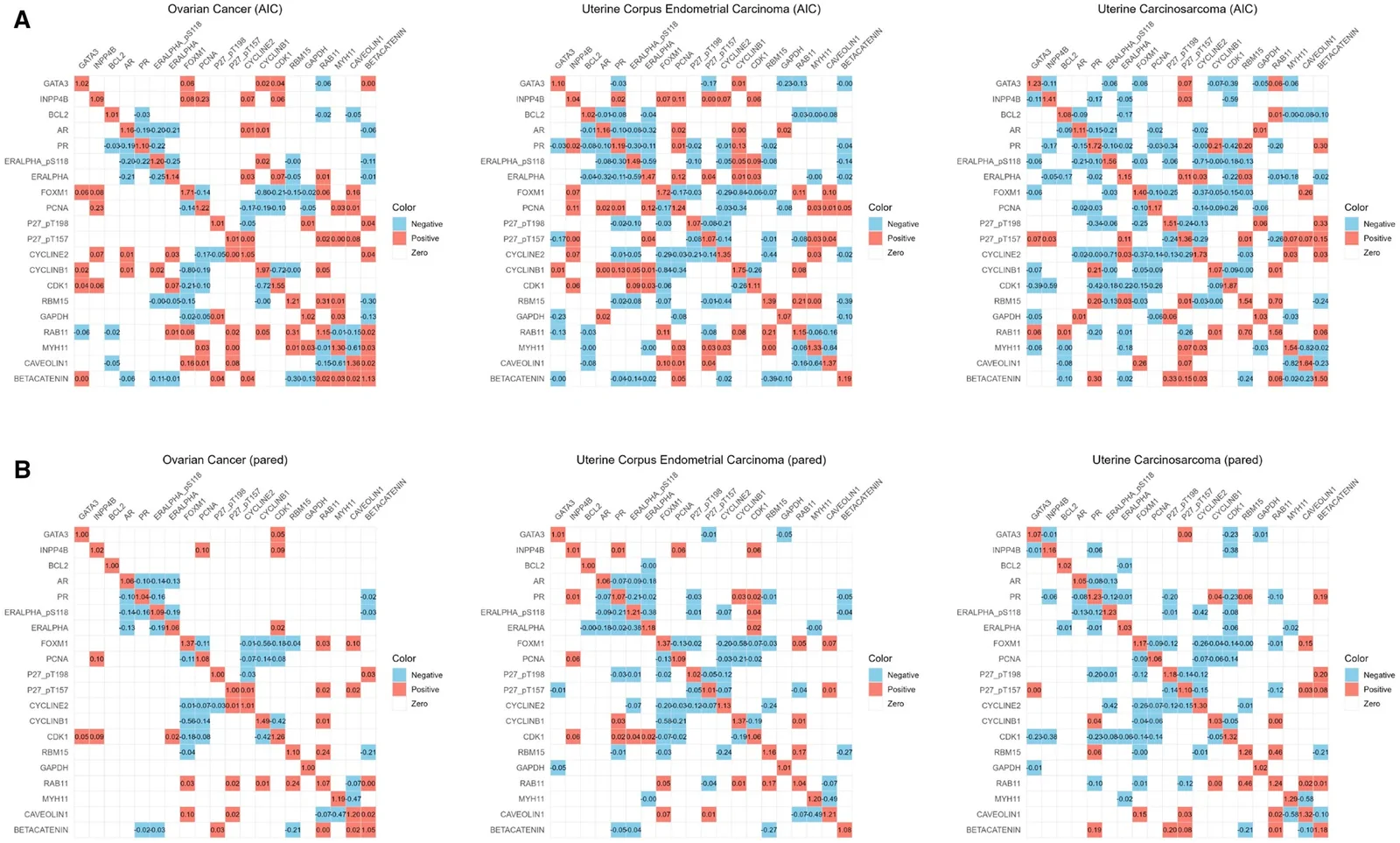
Estimated precision matrices obtained using the group graphical lasso for ovarian (OV), uterine corpus endometrial carcinoma (UCEC), and uterine carcinosarcoma (UCS) for the *breast reactive*, *cell cycle*, *hormone receptor*, and *hormone signaling breast* pathways. (a) shows the network corresponding to the model with minimum AIC. (b) displays a sparser network obtained using pared, representing one of the Pareto-optimal solutions.

We next illustrate the utility of pared in selecting a parsimonious set of protein-signaling networks across gynecological cancer types. To conduct this analysis, we obtained normalized protein abundance data from The Cancer Proteome Atlas (TCPA, Li *et al*. 2013a), which quantifies protein markers using antibodies targeting key oncogenic and cellular signaling pathways. Recent studies have highlighted that similar pathway activities are observed across the gynecological cancer types ovarian (OV), uterine corpus endometrial carcinoma (UCEC), and uterine carcinosarcoma (UCS) (Berger *et al*. 2018). Specifically, the pathways *breast reactive*, *cell cycle*, *hormone receptor*, and *hormone signaling breast* were found to be substantially activated in these cancer types (Das *et al*. 2020). To leverage the potential structural similarity of signaling networks across these cancer types, we applied the group graphical lasso to simultaneously estimate the proteomic networks of OV, UCEC, and UCS using a set of 20 proteins associated with the aforementioned pathways, with sample sizes of 428, 404, and 48, respectively. To illustrate the standard model selection approach, we first applied the AIC, as recommended by Danaher *et al*. (2014). The corresponding estimated precision matrices characterizing the proteomic networks are shown in Fig. 1a.

**Figure 2:**
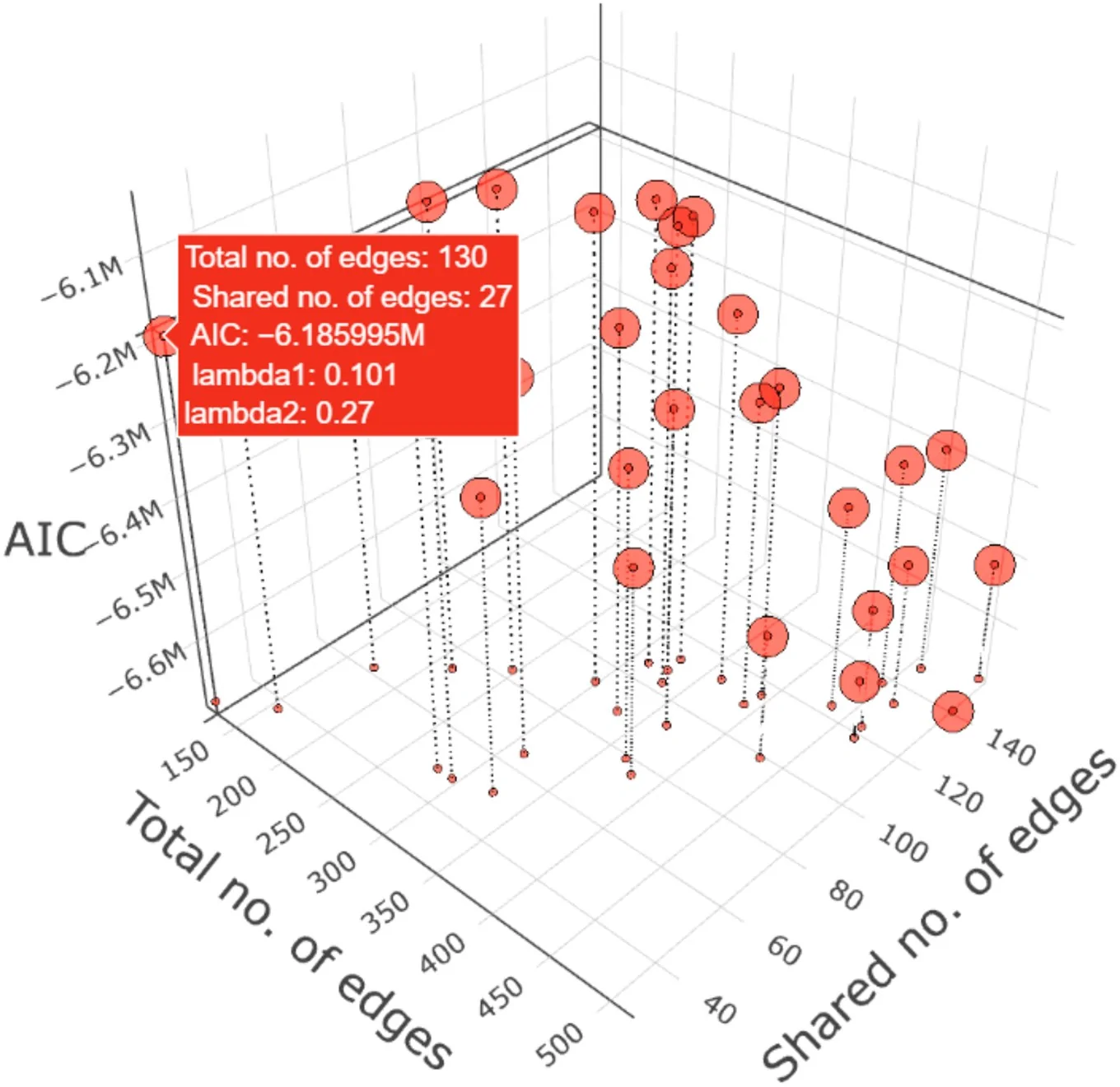
Interactive plot generated using the pared R package, displaying the set of all Pareto-optimal networks obtained by fitting the group graphical lasso to the gynecological cancer proteomic dataset. Hovering over any point reveals the corresponding tuning parameter values and the values of the multi-objective criteria at that solution.

Subsequently, we derived the Pareto-optimal set of models that balance AIC, sparsity, and the number of shared edges across networks using pared, with a run time of 2.4 minutes. Figure 2 presents a screenshot of the interactive Pareto-front plot generated by pared, displaying the optimal set of solution points obtained using the group graphical lasso. Hovering the mouse cursor over any point reveals the corresponding model details, including tuning parameter values and model selection metrics. Figure 1b illustrates one such Pareto-optimal model, which is sparser than the model selected solely based on AIC. This demonstrates the potential of our approach to identify alternative optimal models that are more sparse, offering greater potential for network reliability and interpretability.

## 4 Conclusion

In this work, we introduced pared, a flexible and extensible R package for Pareto-based model selection using multi-objective optimization. Rather than selecting a single tuning configuration by optimizing one criterion, such as cross-validation error or an information criterion, pared returns an estimated Pareto set that allows users to inspect trade-offs among competing model-selection goals. The package is organized around the generic pared_optimize() interface, which permits user-defined tuning parameters, objective functions, objective directions, and solver controls. The initial release also includes convenient built-in wrappers for several common penalized-model settings, including the elastic net, fused lasso, fused graphical lasso, and group graphical lasso.

We demonstrated the utility of pared through both *in silico* validation studies and a real proteomic network analysis. The simulation studies showed that the returned Pareto fronts can contain sparse, truth-compatible models with competitive fit, supporting the use of pared as an exploratory model-selection tool rather than a single automatic selector. In the TCPA case study, pared identified proteomic network models across gynecological cancers that balanced AIC, sparsity, and shared structure, including alternatives that were more parsimonious than the AIC-selected network. The interactive visualization tools further support user-guided exploration of such trade-offs.

Overall, pared provides a reproducible framework for bringing Pareto-front exploration into statistical model selection. The current release focuses on selected penalized regression and graphical-model workflows, but the modular design allows future extensions to additional model classes and objective functions. The same modularity also provides a practical route for incorporating objective-space preferences: as illustrated in the package vignette using a support-vector-machine example, users can modify the objective function so that candidate models exceeding prespecified thresholds, such as model complexity, support-vector fraction, or runtime, receive unfavorable objective values and are therefore discouraged from the returned Pareto set. More systematic approaches to constrained Pareto optimization and preference modeling represent useful directions for future development. In this sense, pared is intended both as a practical software tool for current applications and as a foundation for broader Pareto-based model-selection workflows in statistical learning and computational biology.

## Supplementary Material

vbag211_Supplementary_Data

## Data Availability

The data underlying this article are available at https://github.com/priyamdas2/pared
