# Supplementary material for: pared : model selection using multi-objective optimization
Source: Bioinform Adv. 2026 Aug 19;6(1):vbag211. doi: 10.1093/bioadv/vbag211 (PMC13501534; doi:10.1093/bioadv/vbag211)
Supplement: Supplementary file 1 [file vbag211_supplementary_data.pdf]

Supplementary Material for "pared: Model selection using multi-objective optimization"

Priyam Das*[1], Sarah Robinson[2], and Christine B. Peterson[2]

[1]Department of Biostatistics, Virginia Commonwealth University
[2]Department of Statistics, Rice University

S1 Additional notation and objective definitions

This section provides additional notation for the model equations and objective functions used in the built-in `pared` wrappers. The generic interface `pared_optimize()` can be used with arbitrary user-defined objectives; the definitions below correspond to the default objectives implemented in the initial release of the package.

S1.1 General Pareto-optimization notation

Let $\boldsymbol{x} \in \mathcal{X} \subset \mathbb{R}^d$ denote a vector of tuning parameters. For example, $\boldsymbol{x}$ may contain penalty parameters such as (α, λ) for elastic net regression or (λ_1, λ_2) for joint graphical lasso. Let

$$g(\boldsymbol{x}) = g_1(\boldsymbol{x}), \ldots, g_q(\boldsymbol{x})$$

denote the vector of objective values evaluated after fitting the model at tuning parameter value $\boldsymbol{x}$. Each objective $g_r(\boldsymbol{x})$ may represent a measure of fit, sparsity, smoothness, shrinkage, structural similarity, or computational cost. Objectives may be declared as minimized or maximized. Maximization objectives are internally sign-reversed by `pared` so that Pareto optimization is conducted on a common minimization scale.

A tuning configuration $\boldsymbol{x}_a$ dominates another configuration $\boldsymbol{x}_b$ if $\boldsymbol{x}_a$ is at least as good as $\boldsymbol{x}_b$ in every objective and strictly better in at least one objective, after accounting for the objective directions. The non-dominated tuning configurations define the estimated Pareto set, and their corresponding objective values define the estimated Pareto front.

S1.2 Regression-model notation

For regression problems, let $\mathbf{X} \in \mathbb{R}^{n \times p}$ denote the design matrix, $\mathbf{y} \in \mathbb{R}^n$ the response vector, and $\boldsymbol{\beta} \in \mathbb{R}^p$ the regression coefficient vector. The jth coefficient is denoted by β_j. The number of selected variables is computed as

$$\#j : |\hat{\beta}_j| > \epsilon,$$

where ϵ is a small numerical threshold used to distinguish nonzero coefficients from numerical zeros.

*Corresponding author: dasp4@vcu.edu

S1.2.1 Elastic net

The elastic net combines ℓ_1 and ℓ_2 penalties and estimates $\boldsymbol{\beta}$ by solving

$$\hat{\boldsymbol{\beta}} \underset{\boldsymbol{\beta}}{\operatorname{argmin}} \left[\frac{1}{2n} |\mathbf{y} - \mathbf{X}\boldsymbol{\beta}|_2^2 + \lambda \left\{ \alpha |\boldsymbol{\beta}|_1 + (1-\alpha)|\boldsymbol{\beta}|_2^2 \right\} \right], \tag{1}$$

where $\lambda \geq 0$ controls the overall strength of regularization and $\alpha \in [0,1]$ controls the balance between the ℓ_1 and ℓ_2 penalties. The default `pared_ENet()` objectives are:

- Fit: model deviance, minimized.
- Sparsity: number of nonzero estimated coefficients, minimized.
- Shrinkage: $|\hat{\boldsymbol{\beta}}|_2$, minimized.

The deviance objective measures model fit, the number of nonzero coefficients measures model sparsity, and the ℓ_2 norm measures the overall magnitude of the fitted coefficient vector.

S1.2.2 Fused lasso

The fused lasso adds a penalty on successive coefficient differences and estimates $\boldsymbol{\beta}$ by solving

$$\hat{\boldsymbol{\beta}} = \underset{\boldsymbol{\beta}}{\operatorname{argmin}} \left[\frac{1}{2n} |\mathbf{y} - \mathbf{X}\boldsymbol{\beta}|_2^2 + \lambda_1 |\boldsymbol{\beta}|_1 + \lambda_2 \sum_{j=2}^{p} |\beta_j - \beta_{j-1}| \right], \tag{2}$$

where $\lambda_1 \geq 0$ controls coefficient sparsity and $\lambda_2 \geq 0$ controls smoothness across ordered coefficients. The default `pared_FLasso()` objectives are:

- Fit: residual sum of squares, minimized.
- Sparsity: number of nonzero estimated coefficients, minimized.
- Roughness: mean absolute difference of consecutive estimated coefficients, minimized.

The roughness objective is defined as

$$\frac{1}{p-1} \sum_{j=2}^{p} |\hat{\beta}_j - \hat{\beta}_{j-1}|.$$

This objective favors smoother coefficient profiles across the pre-specified ordering of variables.

S1.3 Graphical-model notation

For graphical-model problems, suppose data are observed for K groups. Let n_k denote the sample size in group k, $\mathbf{S}^{(k)}$ the empirical covariance matrix for group k, and $\boldsymbol{\Theta}^{(k)}$ the corresponding precision matrix. The entry $\theta_{ij}^{(k)}$ denotes the (i,j) element of $\boldsymbol{\Theta}^{(k)}$. Nonzero off-diagonal entries of $\boldsymbol{\Theta}^{(k)}$ correspond to selected edges in the group-specific graphical model. In implementation, an edge is treated as selected when $|\hat{\theta}_{ij}^{(k)}| > \epsilon$ for a small numerical threshold ϵ.

The joint graphical lasso estimates $\boldsymbol{\Theta}^{(k)} * k = 1^K$ by maximizing

$$\{\hat{\boldsymbol{\Theta}}^{(k)}\}_{k=1}^K = \underset{\boldsymbol{\Theta}^{(k)}{}_{k=1}^K}{\operatorname{argmax}} \left[\sum_{k=1}^{K} n_k \left\{ \log\det\left(\boldsymbol{\Theta}^{(k)}\right) \operatorname{tr}\left(\mathbf{S}^{(k)}\boldsymbol{\Theta}^{(k)}\right) \right\} P\left(\{\boldsymbol{\Theta}^{(k)}\}_{k=1}^K\right) \right], \tag{3}$$

over positive-definite precision matrices. Here, $P(\cdot)$ denotes a penalty function that controls sparsity and cross-group similarity.

S1.3.1 Fused graphical lasso

The fused graphical lasso penalty is

$$P(\boldsymbol{\Theta}) = \lambda_1 \sum_{k=1}^{K} \sum_{i \neq j} |\theta_{ij}^{(k)}| + \lambda_2 \sum_{k<k'} \sum_{i,j} |\theta_{ij}^{(k)} - \theta_{ij}^{(k')}|. \tag{4}$$

The first term encourages within-group sparsity, while the second encourages similar edge values across groups. The default `pared_JGL()` objectives with `method = "fused"` are:

- Fit: Akaike information criterion, minimized.
- Sparsity: total number of selected edges across groups, minimized.
- Cross-group similarity: mean absolute difference of estimated precision matrices across groups, minimized.

The total number of selected edges is

$$\sum_{k=1}^{K} \sum_{1 \leq i < j \leq p} I\left(|\hat{\theta}_{ij}^{(k)}| > \epsilon\right).$$

The precision-MAD objective is computed as an average absolute difference in estimated precision-matrix entries across groups, with smaller values indicating greater similarity in edge weights.

S1.3.2 Group graphical lasso

The group graphical lasso penalty is

$$P(\boldsymbol{\Theta}) = \lambda_1 \sum_{k=1}^{K} \sum_{i \neq j} |\theta_{ij}^{(k)}| + \lambda_2 \sum_{i \neq j} \left(\sum_{k=1}^{K} (\theta_{ij}^{(k)})^2\right)^{1/2}. \tag{5}$$

The first term encourages within-group sparsity, while the second encourages shared edge selection across groups. The default `pared_JGL()` objectives with `method = "group"` are:

- Fit: Akaike information criterion, minimized.
- Sparsity: total number of selected edges across groups, minimized.
- Shared structure: number of edges selected in all groups, maximized.

The number of shared edges is

$$\sum_{1 \leq i < j \leq p} I\left\{|\hat{\theta}_{ij}^{(1)}| > \epsilon, \ldots, |\hat{\theta}_{ij}^{(K)}| > \epsilon\right\}.$$

This objective is maximized because larger values correspond to greater overlap in selected network structure across groups.

S1.4 Interpretation of default objectives

Across all built-in wrappers, the default objectives are chosen to represent three broad model-selection goals: fit, parsimony, and structure or regularity. Fit objectives quantify how well the model explains the observed data. Parsimony objectives favor fewer selected variables or edges. Structure or regularity objectives are model-specific: shrinkage for elastic net, smoothness for fused lasso, similarity of edge weights for fused graphical lasso, and overlap of edge selection for group graphical lasso. These default objectives are intended as reproducible templates and can be replaced by user-defined objectives through `pared_optimize()`.

S2 In-silico validation studies

To evaluate `pared` in settings with known ground truth, we conducted two in-silico studies corresponding to two built-in model classes. The first study considers elastic-net regression with a known sparse coefficient vector, allowing assessment of variable-selection behavior. The second study considers group graphical lasso estimation with known precision-matrix structure, allowing assessment of edge-recovery behavior. Because `pared` returns a Pareto set rather than a single automatically selected model, performance was evaluated using both non-oracle representative rules and Pareto-set diagnostics. The non-oracle representatives illustrate how a user may select an interpretable model after inspecting the front, whereas the oracle summaries use the known truth only to assess whether the estimated Pareto set contains truth-compatible solutions.

S2.1 Elastic-net variable-selection simulation

We first evaluated `pared` in a controlled elastic-net regression setting with known sparse truth. The purpose of this simulation was not to show that Pareto-based model selection returns a single universally optimal model. Instead, because `pared` returns a set of non-dominated tuning configurations, the goal was to assess whether the estimated Pareto set contains scientifically useful trade-offs between prediction accuracy, sparsity, and shrinkage. We therefore report both conventional single-rule elastic-net selectors and representative summaries of the `pared` Pareto set.

For each replicate, we generated independent training and test datasets with $n_{\text{train}} = 200$ and $n_{\text{test}} = 200$ observations and $p = 20$ predictors. The predictors followed a mean-zero multivariate normal distribution with a block-correlation covariance structure. Specifically, the variables were divided into four blocks of size five, with within-block correlation $\rho = 0.65$ and zero correlation across blocks. The true regression coefficient vector $\boldsymbol{\beta}_0$ had six nonzero entries, with active predictors indexed by $\{1, 2, 6, 7, 11, 16\}$ and corresponding coefficients $(2.0, 1.5, -2.0, -1.5, 1.75, -1.75)$. The response was generated according to

$$y_i = \mathbf{x}_i^\top \boldsymbol{\beta}_0 + \varepsilon_i, \qquad \varepsilon_i \sim N(0, \sigma^2),$$

where σ^2 was chosen to yield a signal-to-noise ratio of 2.5. Predictors in the test set were standardized using the training-set means and standard deviations. We repeated the experiment for 10 simulation replicates.

We compared `pared` with three conventional elastic-net tuning rules. The first comparator, CV-min, selected the tuning parameters with minimum five-fold cross-validation mean squared error. The second, CV-1SE, selected the sparsest model whose cross-validation error was within one standard error of the minimum. The third, EBIC-min, selected the model minimizing the extended Bayesian information criterion over the same elastic-net tuning grid, using EBIC parameter $\gamma = 0.5$.

The tuning grid used $\alpha \in \{0, 0.1, \ldots, 1\}$, with the corresponding ddefault λ sequence generated by `glmnet`.

For `pared`, the tuning vector was $\mathbf{x} = (\alpha, \log_{10} \lambda)$, with search bounds $\alpha \in [0, 1]$ and $\log_{10} \lambda \in [-3, 1]$. The optimization budget was set to 50 evaluations. For each candidate tuning configuration, `pared` optimized three objectives: the number of nonzero coefficients, the ℓ_2 norm of the fitted coefficient vector, and the model deviance. All three objectives were minimized. After obtaining the Pareto set, we summarized it using two representative rules. The first, `pared` sparse within 10% CV MSE, selected the sparsest Pareto model whose cross-validation MSE was within 10% of the best cross-validation MSE observed on the Pareto set. This is a non-oracle representative that mimics how a user might choose an interpretable model after inspecting the Pareto front. The second, `pared` oracle best F1 on front, selected the Pareto model with the highest variable-selection F1 score, defined as

$$\mathrm{F1} = \frac{2\,\mathrm{TP}}{2\,\mathrm{TP} + \mathrm{FP} + \mathrm{FN}},$$

where TP, FP, and FN denote the numbers of true-positive, false-positive, and false-negative selected variables, respectively. Equivalently, F1 is the harmonic mean of precision, $\mathrm{TP}/(\mathrm{TP} + \mathrm{FP})$, and recall or true-positive rate, $\mathrm{TP}/(\mathrm{TP} + \mathrm{FN})$. This row uses the known simulation truth and is reported only to evaluate whether the Pareto set contains truth-compatible models; it is not a real-data selection rule. For each fitted model, we computed test mean squared error, cross-validation mean squared error, model size, false positives, false discovery rate, true positive rate, F1 score, and the ℓ_2 norm of the coefficient vector. True positives were defined as selected variables belonging to the active set, and false positives were selected variables outside the active set. The F1 score was computed as the harmonic mean of precision and recall. Results are reported as mean and standard deviation across replicates.

Table S1 summarizes the elastic-net simulation results. All methods recovered the six truly active predictors in these simulations, so the main differences were driven by the number of false-positive variables retained. CV-min achieved low prediction error but selected substantially larger models, with an average model size of 13.4 and 7.4 false-positive variables. CV-1SE and EBIC-min reduced the number of false positives relative to CV-min, with only moderate changes in prediction error. The non-oracle `pared` sparse representative achieved a comparable prediction-error range while selecting fewer variables than CV-min and reducing the false discovery rate. The oracle best-F1 row shows that the Pareto set contained substantially sparser models with stronger variable-selection performance, achieving an average F1 score of 0.932 and an average false discovery rate of 0.125. As expected, this oracle representative sacrifices prediction error because it is selected for truth recovery rather than prediction accuracy.

Method	Test MSE	CV MSE	Model size	FP	FDR	F1	ℓ_2 norm
CV-min	11.31 (1.47)	11.48 (1.19)	13.4 (2.84)	7.4 (2.84)	0.536 (0.085)	0.629 (0.082)	3.86 (0.238)
CV-1SE	11.69 (1.51)	11.99 (1.26)	8.6 (0.97)	2.6 (0.97)	0.294 (0.080)	0.825 (0.055)	3.52 (0.259)
EBIC-min	11.24 (1.48)	11.59 (1.21)	9.6 (1.43)	3.6 (1.43)	0.363 (0.088)	0.775 (0.067)	3.71 (0.167)
pared sparse within 10% CV MSE	11.83 (1.50)	12.19 (1.12)	9.4 (1.27)	3.4 (1.27)	0.352 (0.084)	0.784 (0.062)	3.42 (0.201)
pared oracle best F1 on front	17.61 (3.17)	18.09 (4.36)	6.9 (0.57)	0.9 (0.57)	0.125 (0.074)	0.932 (0.041)	2.39 (0.478)

Table S1: Elastic-net simulation results across 10 replicates. Values are mean (SD). All methods recovered all six active predictors in these simulations; therefore, TP and TPR are omitted from the display table. The best-F1 `pared` row uses the known truth and is reported only to assess whether the Pareto front contains truth-compatible models.

Overall, this simulation illustrates the intended role of `pared`: rather than replacing cross-validation with a single automatic tuning rule, it exposes a Pareto front containing models with different fit-sparsity-shrinkage trade-offs. The non-oracle sparse representative provides a practical example of selecting a parsimonious model with competitive prediction performance, while the oracle front-level summary confirms that the Pareto set can contain models close to the known sparse truth.

S2.2 Group graphical lasso edge-recovery simulation

We next evaluated `pared` in an in-silico group graphical lasso (GGL) setting with known precision-matrix structure. This example was designed to complement the elastic-net variable-selection simulation by considering a network-estimation problem in which model selection must balance goodness-of-fit, sparsity, and shared graphical structure across groups. As with the elastic-net simulation, the goal was not to force `pared` to return a single universally optimal model, but rather to evaluate whether the estimated Pareto front contained useful trade-offs under known truth.

We generated data for $K = 4$ groups with $p = 20$ variables and group sample sizes $(100, 200, 50, 150)$. The first precision matrix was defined as

$$\boldsymbol{\Theta}^{(1)} = \text{toeplitz}(1, 0.5, 0.4, 0, \dots, 0),$$

so that only the first- and second-order off-diagonal bands were nonzero. To generate related but non-identical networks for the remaining groups, we started from the previous group's precision matrix and progressively perturbed the graph structure. Specifically, for group 2, five randomly selected nonzero upper-triangular edges from $\boldsymbol{\Theta}^{(1)}$ were removed and five randomly selected zero edges were added with weights drawn uniformly from $[0.4, 0.6]$ and assigned random signs. For group 3, ten additional edge deletions and additions were applied to the group-2 matrix, and for group 4, five additional deletions and additions were applied to the group-3 matrix. The random seed was fixed at 1. The resulting matrices were adjusted to be positive definite using the same correction routine used in the package implementation. For each group k, observations were then generated independently from a multivariate normal distribution with mean zero and covariance matrix $(\boldsymbol{\Theta}^{(k)})^{-1}$.

We compared `pared` with an AIC-selected GGL baseline. The baseline fit group graphical lasso models over a two-dimensional grid, with $\log_{10}(\lambda_1)$ and $\log_{10}(\lambda_2)$ each evaluated on an equally spaced grid from -2 to 0, and selected the model with the smallest AIC. For `pared`, the tuning vector was $\mathbf{x} = (\log_{10} \lambda_1, \log_{10} \lambda_2)$, with the same search bounds $[-2, 0] \times [-2, 0]$ and an optimization budget of 100 evaluations. The three objectives were AIC, total number of selected edges across groups, and number of shared edges across all groups. AIC and total edges were minimized, whereas shared edges were maximized.

After obtaining the Pareto set, we summarized it using three representative rules. The first, `pared` sparse GGL within 10% AIC, selected the Pareto model with the fewest total edges among models whose AIC was within 10% of the best Pareto AIC. The second, `pared` shared-edge GGL within 10% AIC, selected the Pareto model with the largest number of shared edges under the same AIC tolerance. The third, `pared` best-F1 GGL on front, selected the Pareto model with the highest edge-recovery F1 score. This final row uses the known simulation truth and is reported only as a Pareto-set diagnostic, not as a real-data selection rule.

For each fitted model, we computed AIC, total selected edges across groups, number of shared edges, true positives, false positives, false discovery rate (FDR), true-positive rate (TPR), F1 score, and runtime. Edges were evaluated using the upper triangular entries of the group-specific precision matrices and compared with the known nonzero edge structure. Results are shown in Table S2. The

Method	AIC	Total edges	Shared edges	TP	FP	FDR	TPR	F1
AIC-selected GGL	13194.44	504	45	143	361	0.716	0.966	0.439
`pared` sparse GGL within 10% AIC	14476.92	198	23	110	88	0.444	0.743	0.636
`pared` shared-edge GGL within 10% AIC	13308.10	712	146	144	568	0.798	0.973	0.335
`pared` best-F1 GGL on front	14476.92	198	23	110	88	0.444	0.743	0.636

Table S2: Group graphical lasso simulation results for one in-silico dataset with known precision-matrix structure. The `pared` rows summarize different representatives of the estimated Pareto front. The best-F1 row uses the known truth and is reported only to assess whether the Pareto front contains truth-compatible network estimates. In this example, the sparse `pared` representative and the best-F1 Pareto representative selected the same model.

AIC-selected GGL baseline achieved the smallest AIC but selected a dense model, with 504 total edges and 361 false-positive edges. The sparse `pared` representative selected substantially fewer edges, reducing the total edge count from 504 to 198 and the number of false-positive edges from 361 to 88. This reduction led to a lower FDR, decreasing from 0.716 to 0.444, and a higher F1 score, increasing from 0.439 to 0.636. In this example, the sparse `pared` representative also coincided with the best-F1 model on the Pareto front, indicating that the front contained a substantially more truth-compatible network than the AIC-selected baseline. The shared-edge representative, by contrast, emphasized common structure across groups and selected 146 shared edges, but at the cost of a much denser and less accurate network. This illustrates the intended role of `pared`: different Pareto-front representatives expose different scientific trade-offs, allowing users to choose among fit, sparsity, and shared structure rather than relying on a single scalar criterion.

S3 Computation times

In the accompanying GitHub vignette[1], we present a set of example scenarios in which Pareto-optimal models are identified using the `pared` R package, fitting elastic net regression, fused lasso regression, and joint graphical lasso with both *fused* and *group* penalties. All computations are performed on a desktop running the Windows 10 Enterprise operating system desktop with 32 GB RAM and the following processor characteristics: 12th Gen Intel(R) Core(TM) i7-12700, 2100 Mhz, 12 Cores(s), 20 Logical Processor(s). The computation times and other model parameter details are provided below in Table S3. The parameter `Pareto_budget`, set by the user, denotes the maximum number of objective function evaluations allowed during the multi-objective optimization (MOO) process within `pared` functions.

R functions	Model	Parameters	Sample size	Pareto Budget	Computation time (sec.)
`pared_ENet`	Elastic net	$p = 5$	100	50	83.91
`pared_FLasso`	Fused lasso	$p = 10$	100	80	140.47
`pared_JGL`	Fused JGL	$K = 4, p = 20$	(20, 40, 60, 80)	40	131.31
`pared_JGL`	Group JGL	$K = 4, p = 20$	(30, 50, 40, 70)	50	83.40

Table S3: Computation times (in seconds) required to identify the optimal set of Pareto-optimal models using the `pared` R package functions: `pared_ENet`, `pared_FLasso`, and `pared_JGL`.

For the case study involving proteomic networks (each of size 20) for ovarian (OV), uterine corpus endometrial carcinoma (UCEC), and uterine carcinosarcoma (UCS) cancers, with sample

[1] `https://github.com/priyamdas2/pared`

sizes of 428, 404, and 48, respectively, it took 141 seconds (2.4 minutes) to estimate the Pareto-front curve using the `pared_JGL` function with the *group* penalty.
